# FAIR in action: Brain-CODE - A neuroscience data sharing platform to accelerate brain research

**DOI:** 10.3389/fninf.2023.1158378

**Published:** 2023-05-18

**Authors:** Brendan Behan, Francis Jeanson, Heena Cheema, Derek Eng, Fatema Khimji, Anthony L. Vaccarino, Tom Gee, Susan G. Evans, F. Chris MacPhee, Fan Dong, Shahab Shahnazari, Alana Sparks, Emily Martens, Bianca Lasalandra, Stephen R. Arnott, Stephen C. Strother, Mojib Javadi, Moyez Dharsee, Kenneth R. Evans, Kirk Nylen, Tom Mikkelsen

**Affiliations:** ^1^Ontario Brain Institute, Toronto, ON, Canada; ^2^Datadex, Toronto, ON, Canada; ^3^Indoc Research, Toronto, ON, Canada; ^4^Rotman Research Institute, Toronto, ON, Canada; ^5^Department of Pharmacology and Toxicology, University of Toronto, Toronto, ON, Canada

**Keywords:** neuroinformatics, neuroscience, data sharing, data management, FAIR

## Abstract

The effective sharing of health research data within the healthcare ecosystem can have tremendous impact on the advancement of disease understanding, prevention, treatment, and monitoring. By combining and reusing health research data, increasingly rich insights can be made about patients and populations that feed back into the health system resulting in more effective best practices and better patient outcomes. To achieve the promise of a learning health system, data needs to meet the FAIR principles of findability, accessibility, interoperability, and reusability. Since the inception of the Brain-CODE platform and services in 2012, the Ontario Brain Institute (OBI) has pioneered data sharing activities aligned with FAIR principles in neuroscience. Here, we describe how Brain-CODE has operationalized data sharing according to the FAIR principles. Findable—Brain-CODE offers an interactive and itemized approach for requesters to generate data cuts of interest that align with their research questions. Accessible—Brain-CODE offers multiple data access mechanisms. These mechanisms—that distinguish between metadata access, data access within a secure computing environment on Brain-CODE and data access via export will be discussed. Interoperable—Standardization happens at the data capture level and the data release stage to allow integration with similar data elements. Reusable - Brain-CODE implements several quality assurances measures and controls to maximize data value for reusability. We will highlight the successes and challenges of a FAIR-focused neuroinformatics platform that facilitates the widespread collection and sharing of neuroscience research data for learning health systems.

## Introduction

The sharing of data in the health biosciences domain is a vital component of advancing scientific research and accelerating discovery—with several funding agencies now mandating the sharing of datasets for such purposes (National Institutes of Health, 2023 NIH Data Management and Sharing Policy, Wellcome Trust Data (2017), Software and Materials Management and Sharing Policy, Government of Canada (2021) Tri-Agency Research Data Management Policy). The ability to harness knowledge from such datasets is dependent on there being sufficient information available that document their creation and curation. This is particularly important in a learning health system where research findings can be used to inform clinical care in the future. Four foundational principles of data sharing—Findability, Accessibility, Interoperability, Reusability (FAIR)—have emerged in the last decade as guiding elements on how datasets should be structured, annotated, and packaged to enable maximal reuse (Wilkinson et al., 2016). Within the domain of neuroscience, there has been movement toward greater efforts around data standardization in alignment with the FAIR principles (Poline et al., 2022).

The Ontario Brain Institute (OBI) is a provincially funded, not-for-profit organization founded in 2010 that accelerates discovery and innovation, benefiting both patients and the economy (Stuss, 2014). OBI funds research activities across several neuroscience domains through its Integrated Discovery Program (IDP) model. These pan-Ontario programs take an approach to research that spans several disciplines and brings together a diverse group of stakeholders including researchers, clinicians, industry partners, and patients and their advocates. Programs collect multiple data types including, but not limited to clinical, imaging, and molecular data. Within their studies, the programs have also incorporated standardized consent language to allow for re-use of de-identified datasets by external researchers and organizations (Lefaivre et al., 2019). This consent language was developed, in consultation with provincial research ethics board chairs and the Information and Privacy Commissioner of Ontario, in 2015 to support data sharing both within IDPs, as well as with external researchers and organizations. The consent language also speaks to linkage of data sets with independent databases, how participant information is kept confidential, and how participants can request withdrawal of data from respective studies.

To support the activities of these IDPs, a large-scale neuroinformatics platform—Brain-CODE—was developed to support the collection, storage, federation, sharing and analysis of different data types across several brain disorders. The technical and governance features of Brain-CODE have been previously described (Vaccarino et al., 2018; Lefaivre et al., 2019). This article will focus on the data sharing processes on Brain-CODE and their alignment with the FAIR principles.

## Alignment with FAIR principles— Findability

**FIGURE 1:**
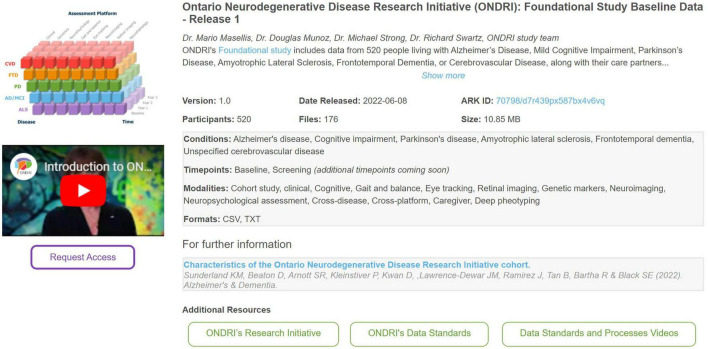
An example of how information about a data release is presented to a data requestor on the Brain-CODE platform—the Ontario neurodegenerative disease research initiative (ONDRI): Foundational study baseline data—Release 1 (Farhan et al., 2017; Sunderland et al., 2023).

All available datasets for re-use are highlighted on the Brain-CODE portal.^1^ An important element of Findability relates to describing datasets with rich and concise metadata. As such, Brain-CODE presents each data release with an initial description followed by standardized metadata including version #, data release date, # of participants, # of files, and overall dataset size. Other information presented about each data release include conditions of interest standardized to the Medical Dictionary for Regulatory Activities (MedDRA) ontology,^2^ imaging scan type including task type described within The Cognitive Atlas knowledge base,^3^ as well as data collection timepoints, modalities, and file formats. Figure 1 highlights how a data release is typically presented to a data requestor.

As part of their activities, IDPs also highlight the availability of their datasets through their respective presentations and publications. Relatedly, OBI has partnered with the Canadian Open Neuroscience Platform (CONP) in the advertising of available datasets on the Brain-CODE portal. The CONP is a national network of Canadian neuroscience research centers committed to collaborating on a series of new open neuroscience initiatives (Harding et al., 2022).^4^ All Brain-CODE data releases are registered on the searchable CONP data portal (Poline et al., 2023) and are described according to a customized version of the Data Tags Suite (DATS) metadata model (Alter et al., 2020). Another key feature of the Findability Principle is the assignment of a globally unique and persistent identifier to the respective dataset. Via involvement in CONP, Brain-CODE datasets are assigned an Archival Resource Key (ARK) ID^5^ –a persistent identifier for information objects. In the near future, Brain-CODE plans to incorporate Digital Object Identifiers (DOIs) linked to their respective data releases.

Further advances are being planned to enhance Brain-CODE’s Findability including expanding upon the current study-specific data release configuration to allow for cross-study query and cohort creation. This will enable data requestors to pool datasets across respective studies for integrative analyses. Additionally, OBI will continue to enhance the findability of datasets on the Brain-CODE portal through the incorporation of standardized metadata schemas (e.g., schema.org) to allow for querying by certain data search engines (e.g., Google Dataset Search). Finally, OBI is a member of the Global Alliance for Genomics and Health (GA4GH)–a policy-framing and technical standards-setting organization, seeking to enable responsible genomic data sharing (Terry, 2014)—and OBI continues to examine the incorporation of tools from various GA4GH driver projects [e.g., tagging of usage restrictions linked to datasets via the GA4GH Data Use Ontology (DUO)] (Lawson et al., 2021).

## Alignment with FAIR principles—Accessibility

There are different data access mechanisms based on the type of data that are being requested. Before data can be requested for access, there is a 12-month exclusivity period during which IDPs maintain exclusive access to their data. Data is then made accessible via either a Public or Controlled Access Mechanism on Brain-CODE. Datasets that have not previously contained Personal Health Information Protection Act [PHIPA] (2004) are made available by the Public Access Mechanism and can be accessed via the Brain-CODE portal without submitting a data access request proposal. Once a Brain-CODE account is created, data requestors utilize interactive dashboards to explore the data and metadata, select packages of interest, and download their respective data cuts. The most recent Public Access data release on Brain-CODE involves a priority setting partnership for epilepsy and seizures that was conducted by OBI, its epilepsy research program, EpLink, and the James Lind Alliance.^6^

**FIGURE 2:**
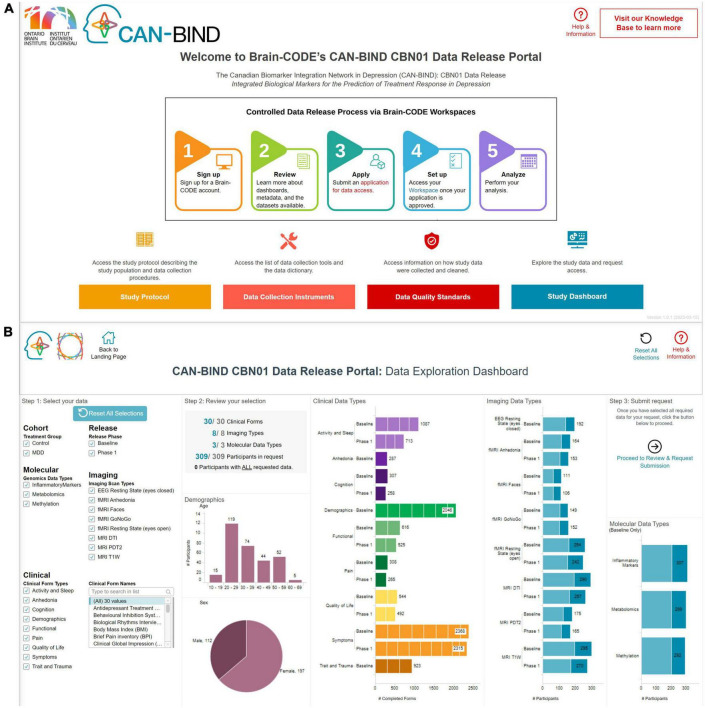
An example of how a data release is presented to the data requestor in terms of **(A)** providing supporting documentation [top figure—i.e., study protocol (Orange), data collection instruments (Pink), data quality standards (Red)] and **(B)** how interactive visualizations built using provided metadata can be used to select data packages of interest—CAN-BIND integrated biological markers for the prediction of treatment response in depression data release.

Datasets that have previously contained PHI are made available by the Controlled Access Mechanism. A recent Controlled Access Mechanism data release is from the Canadian Biomarker Integration Network in Depression (CAN-BIND) and its foundational study (Lam et al., 2016; Figure 2). To prepare datasets to be released through this mechanism, IDPs provide data files, modality specific data dictionaries, and README files which are used to create an interactive Data Release Dashboard through which data requests can be submitted. Data files and supplementary documentation are manually reviewed by the IDP and OBI for direct identifiers as defined in OBI’s Brain-CODE Governance policy,^7^ such that the data are suitably de-identified (Theyers et al., 2021) prior to being available for external requests. The selection of these direct identifiers stems from legislation governing Brain-CODE activities in Ontario, Canada, notably the Personal Health Information Protection Act [PHIPA] (2004). While PHIPA does provide a definition of de-identification, there is limited guidance on its implementation. As such, OBI looked to methods in other jurisdictions, namely the U.S. Safe Harbor provision of the U.S. Health Information Portability and Accountability Act [HIPAA] (1996), and incorporated and customized such processes to both reduce risk of re-identification while ensuring usability of data sets made available via Brain-CODE.

Data requestors can review available data and metadata, select data packages of interest, and then submit a Data Access Request through the study Data Release Dashboard on the Brain-CODE portal. The data requestor will also be expected to provide further information about their planned analyses, provide documentation that an ethics committee has reviewed the project, and sign a Data Use Agreement. All requests are reviewed by the Brain-CODE Data Access Committee (DAC), composed of representatives from IDPs, experts in data privacy, and community representatives. The DAC provides a recommendation to approve or reject a data access request to the Brain-CODE Steering Committee, which is composed of OBI executive members. Once approved, access to data is granted either within a secure workspace on Brain-CODE or via local download. For the latter option, a data transfer agreement must be executed. These processes ensure that there are sufficient administrative and technical safeguards in making data sets available to data requestors in a secure manner.

## Alignment with FAIR principles—Interoperability

Perhaps the greatest challenge in harnessing the full value of data is interoperability. While a dataset alone can have great utility in answering multiple questions, the prospect of combining a single dataset with others can increase its value by order of magnitude. Among the most critical aspect of data interoperability is the adoption of common data types, file formats, common data elements, semantic annotations, as well as common data packaging, metadata description, and indexing. Furthermore, platforms that aim to facilitate interoperability should provide the mechanisms and services that facilitate the combination and linking of data in useful ways.

Brain-CODE was designed to capture clinical, imaging, and molecular/genomics data with the help of three distinct but consistently used electronic data capture (EDC) systems, namely REDCap for demographics and clinical data, SPReD based on the XNAT platform for brain imaging data, and LabKey for molecular and genomics data (Vaccarino et al., 2018). The consistent use of EDC systems facilitates the curation and export of data to standard formats. These formats include comma separated value (CSV) files for tabular data, Neuroimaging Informatics Technology Initiative (NIfTI) files for binary imaging data,^8^ the European Data Format (EDF +) files for times-series data,^9^ and text files for applicable molecular and genomic data. Each of these are common non-proprietary file formats that can be used by numerous software for analysis or further processing.

With respect to common data elements (CDEs), OBI realized the opportunity to identify and adopt a common set of measures for demographic and clinical information collection across its IDPs. Established in 2013, CDEs were developed via a Delphi consensus process through the engagement of the clinical and research neuroscientific community among the IDPs. Identified CDEs span nine sub-domains of inquiry including demographics, socioeconomic status, quality of life, activities of daily living, medical comorbidity, psychiatric comorbidity, depression, anxiety, and sleep, and have been utilized for cross-disorder analysis (Vaccarino et al., 2022).

Datasets on Brain-CODE typically originate at the study level and are organized into distinct packages that can be based on modality type (e.g., clinical, imaging, molecular, etc.), modality subtype (e.g., MRI, EEG), participant cohort, and/or study timepoints. For imaging data, elements of the BIDS data structure standard have been adopted to facilitate interoperability of the data with various research software tools and other BIDS datasets as the adoption of this standard becomes increasingly common amongst the brain imaging research community (Gorgolewski et al., 2016). OBI has worked with the IDPs to collect and document metadata, including study protocol details, data collection processes, preprocessing and data provenance information, and details regarding study contributors, publications, etc. In addition, video presentations regarding the respective study have been published to accompany the most recent releases.^10^

To further support the combining of data, Brain-CODE facilitates the linking of data using advanced privacy preserving record linking (PPRL) via a deterministic El Gamal homomorphic encryption and matching based protocol (Gee et al., 2018). By collecting sensitive direct identifiers (such as provincial health card numbers in Ontario, Canada) in an encrypted format, Brain-CODE can match participant records with other data providers to achieve linkages where new information can be added to data available for the same participants while preserving privacy. With this mechanism, OBI data partners such as the Institute for Clinical Evaluative Sciences (ICES) can link health administrative data with research data on Brain-CODE (Behan et al., 2020; Southwell et al., 2022). As a result, rich participant profiles can be generated to answer deeper research questions.

## Alignment with FAIR principles—Reusability

Reusability can only be achieved if the prior three principles are well implemented. Without findability, accessibility, and interoperability, the reuse of data will be challenging. In addition, reusability requires that critical pieces of information accompany datasets and that expert support is provided to data requestors in a consistent and reliable manner with respect to the use of data. While OBI seeks to define upfront standards for datasets, novel data requests may necessitate data to be reformatted, described, or computed in new ways. As a result, OBI seeks to follow key steps for data reuse including simplicity, portability, annotation, and quality reporting.

Simplicity in data formats and packaging help ensure that data requestors will have the ability to interpret and ingest the data using well established processing and analysis tools. As discussed above, common data formats that are non-proprietary, capture essential information, and have the flexibility to be extended are preferred on Brain-CODE. Combining data files with metadata files in a simple data package hierarchy that are consistently implemented across Brain-CODE also plays a role in achieving this.

Portability of data and metadata is essential for nimble reuse of data under various circumstances. For example, data administrators may need to load the data in a secure computing enclave that meets specific analysis protocols. Data may also need to be transformed to match target analytics model needs. If the data were scattered across multiple databases and file systems, it would require significant resources to manage, as well as being more susceptible to errors. By adopting a standard packaging approach based on a hierarchical file system, datasets are portable and can be processed as required to meet the target use case.

Rich annotation is important to generate during data collection and curation to better support data reuse. Fields including their labels, values, and units should be semantically coded according to standard control vocabularies and ontologies to maximize the opportunities for remodeling of data and their structure. For example, a data requestor may require data to match an observation medical outcomes partnership (OMOP) common data model (CDM) to combine with other OMOP CDM data. Without suitable annotations, a mapping from a source structure and labels to OMOP, or to another required data model, cannot be achieved resulting in a failure of data reuse. While rich annotations are important, the process can lead to over-engineered data that negatively impacts simplicity. As a result, Brain-CODE selectively adopts rich semantic vocabularies and data structures on an as-needed basis or as clear standards emerge within a particular domain of research. This is an active area of development for data on Brain-CODE.

Data quality reports provide data requestors with key information on the characteristics of data which helps with the identification of data origin, data completeness, and data integrity. This is a critical element to help create trust in the data and in the veracity of results from their reuse. Brain-CODE generates visual dashboards with rich data characteristics that data requestors benefit from when interpreting the data as we continue to work on improving this quality reporting.

Finally, ongoing support by the Brain-CODE informatics team helps ensure that data requestors understand the steps required for access, in what manner they can access the data (such as download to their local machine or via the use of a computing workspace), and to answer any further questions related to the origin and characteristics of the data. Without a human-in-the-loop, requests can stall and opportunities for discovery may not be realized.

## Discussion

Altogether, Brain-CODE is a functioning example within the neuroscience field as to how re-use of datasets can be supported in alignment with the FAIR principles. To date, Brain-CODE has handled hundreds of data access requests from both academic and non-academic groups globally. This has allowed for greater opportunities for data exploration as well as affording data requestors the opportunity to address research questions of interest without having to initiate large-scale data collection efforts. The Brain-CODE platform continues to be developed to enhance data sharing efforts to allow for greater data discovery and understanding of various brain disorders.

## Data availability statement

The original contributions presented in this study are included in the article/supplementary material, further inquiries can be directed to the corresponding author.

## Author contributions

BB, FJ, and HC wrote the first draft of the manuscript and prepared the manuscript. All authors contributed to the development of the data sharing processes via Brain-CODE and commented on/revised the manuscript at all stages.

## Footnotes

1
www.braincode.ca

2
https://www.meddra.org/

3
https://www.cognitiveatlas.org/

4
https://conp.ca/

5
https://arks.org/

6
https://www.jla.nihr.ac.uk/news/epilepsy-canada-psp-open-dataset-available/30802

7
https://braininstitute.ca/research-data-sharing/brain-code

8
https://nifti.nimh.nih.gov/

9
https://www.edfplus.info/

10
https://www.braincode.ca/content/controlled-data-releases
